# Validity and reliability of a modified english version of the physical activity questionnaire for adolescents

**DOI:** 10.1186/s13690-016-0115-2

**Published:** 2016-01-22

**Authors:** Daniel Aggio, Stuart Fairclough, Zoe Knowles, Lee Graves

**Affiliations:** Department of Epidemiology and Public Health, University College London, London, WC1E6BT UK; Department of Sport and Physical Activity, Edge Hill University, St. Helens Road, Ormskirk, Lancashire L39 4QP UK; Department of Physical Education and Sport Sciences, University of Limerick, Limerick, Ireland; Physical Activity Exchange, Research Institute for Sport and Exercise Sciences, Liverpool John Moores University, 62 Great Crosshall Street, Liverpool, L3 2AT UK

**Keywords:** Physical activity, Self-report, Questionnaires, Accelerometry, Youth, Validity

## Abstract

**Background:**

Adaptation of physical activity self-report questionnaires is sometimes required to reflect the activity behaviours of diverse populations. The processes used to modify self-report questionnaires though are typically underreported. This two-phased study used a formative approach to investigate the validity and reliability of the Physical Activity Questionnaire for Adolescents (PAQ-A) in English youth. Phase one examined test content and response process validity and subsequently informed a modified version of the PAQ-A. Phase two assessed the validity and reliability of the modified PAQ-A.

**Methods:**

In phase one, focus groups (*n* = 5) were conducted with adolescents (*n* = 20) to investigate test content and response processes of the original PAQ-A. Based on evidence gathered in phase one, a modified version of the questionnaire was administered to participants (*n* = 169, 14.5 ± 1.7 years) in phase two. Internal consistency and test-retest reliability were assessed using Cronbach’s alpha and intra-class correlations, respectively. Spearman correlations were used to assess associations between modified PAQ-A scores and accelerometer-derived physical activity, self-reported fitness and physical activity self-efficacy.

**Results:**

Phase one revealed that the original PAQ-A was unrepresentative for English youth and that item comprehension varied. Contextual and population/cultural-specific modifications were made to the PAQ-A for use in the subsequent phase. In phase two, modified PAQ-A scores had acceptable internal consistency (α = 0.72) and test-retest reliability (ICC = 0.78). Modified PAQ-A scores were significantly associated with objectively assessed moderate-to-vigorous physical activity (*r* = 0.39), total physical activity (*r* = 0.42), self-reported fitness (*r* = 0.35), and physical activity self-efficacy (*r* = 0.32) (*p* ≤ 0.01).

**Conclusions:**

The modified PAQ-A had acceptable internal consistency and test-retest reliability. Modified PAQ-A scores displayed weak-to-moderate correlations with objectively measured physical activity, self-reported fitness, and self-efficacy providing evidence of satisfactory criterion and construct validity, respectively. Further testing with more diverse English samples is recommended to provide a more complete assessment of the tool.

**Electronic supplementary material:**

The online version of this article (doi:10.1186/s13690-016-0115-2) contains supplementary material, which is available to authorized users.

## Background

Regular physical activity (PA) in childhood is important for the prevention of several non-communicable disease risk factors [1]. In England, less than a quarter of young people meet the current recommendations of 60 min moderate-to-vigorous PA (MVPA) per day [2] and recent research has observed steep declines in activity levels during adolescence [3]. Accurate measurement of PA is therefore paramount for epidemiological and intervention research. Self-report questionnaires, despite their limitations, are often used within population surveillance studies due to their practicality, low cost, low participant burden and ability to contextualise PA [4]. Objective measures provide a more accurate estimate of PA but do not provide contextual information and can be difficult to administer in youth populations. Self-report questionnaires continue to be an important measure of PA; therefore efforts to improve their ability to accurately capture PA data in youth should be continued for future research.

A systematic review by Chinapaw and colleagues examining the validity, reliability and responsiveness of PA questionnaires in youth found that none of the questionnaires included in the review had acceptable levels of reliability and validity according to guidelines described in the Qualitative Attributes and Measurement Properties of Physical Activity Questionnaires (QAPAQ). [5] The Physical Activity Questionnaire for Adolescents (PAQ-A) [6] is, however, regarded as one of the most suitable self-report tools for examining PA in these populations [7]. It was developed in Canada but has been widely used in other parts of the world, including African countries, such as Nigeria and Ghana [8, 9], in Europe, including Holland and the UK [3, 10], and in specific youth populations, such as adolescents with cerebral palsy [11]. The PAQ-A has been validated against objectively measured PA (*r* = 0.33–0.63) and other self-reported measures of PA (*r* = 0.73) [12–14]. Objective measures of PA, such as accelerometers, are accurate and reliable measures of PA [15] and are commonly used as a criterion reference method for assessing the validity of self-reported PA questionnaires [12–14]. Construct validity of the PAQ for older children (PAQ-C) has been demonstrated through correlations with aerobic fitness (*r* = 0.28) and perceptions of athletic competence (*r* = 0.48) [16]. Acceptable test-retest reliability and internal consistency have also been demonstrated for the PAQ-C and PAQ-A [13, 17]. Furthermore, the PAQ-A has been used to generate a wealth of evidence demonstrating associations between PA and other related constructs, such as fat mass, bone mineral content and cardiorespiratory fitness [18–20].

Despite this level of evidence, the widespread use of and advocacy for the PAQ [21] arguably warrants a stronger level of validity by examining population-specific test content and response processes, which are two key sources of validity [22]. These sources of validity are important for providing evidence that questionnaire items are both representative and understood by respondents. Evidence for test content and response process validity is typically gathered during the instrument development phase; however, it may be important to gather this evidence to inform questionnaire modifications for use in other populations or after significant changes in the popularity of different sports and activities, which can occur over time [23]. During the development of the PAQ-A, Crocker et al. [17] made content modifications based on student and research assistant feedback and item analysis, but comprehensive evidence for test content and response process validity was not reported. Under-representation of PA domains could consequently impair associations with related constructs, which may particularly be an issue when questionnaires are used with different populations. For example, it cannot be assumed that a questionnaire developed and validated in one country is suitable for youth in another country without re-evaluating the appropriateness of questionnaire items [24]. Cross-cultural comparisons have revealed differences in measures of fitness, height, weight and participation in specific types of activities between Canadian and English youth of the same age [25]. Thus, cultural differences as well as changes to the popularity of specific sports and activities have the potential to inhibit questionnaire validity. Previous studies have modified the original PAQ, reflecting activities deemed more representative to the study population [26], but typically decision-making processes used to add or remove activities from the original questionnaire are underreported [14, 27] or not reported at all [28]. Detailing such processes will provide a more transparent procedure for modifying questionnaires. Thus, the aims of this study were (1) to use formative focus groups to inform modifications to the PAQ-A for use with the English adolescents, and (2) to investigate the reliability and validity of the modified PAQ-A.

## Methods

### Study design

The study was conducted in two phases. Phase one used qualitative techniques to assess the test content and response process validity of the PAQ-A, which informed potential modifications required prior to phase two. Phase two assessed the validity and reliability of the modified version of the PAQ-A. Ethical approval was granted by University Research Ethics Committee.

### Phase one

#### Participants

Three secondary schools in North West England were invited to participate in phase one of the study. Schools participating were typical of schools in the North West in terms of size and academic performance. Participants (*n* = 20) were aged between 13 and 16, covering the majority of secondary school year groups in compulsory education. All participants provided written informed parental consent and assent.

#### Focus groups

Focus groups and observing/interviewing respondents have previously been employed to obtain evidence for test content and response processes [29], and are deemed appropriate methods for exploring the thoughts and feelings of young people [30]. The focus group questions were designed to flow from completion of the PAQ-A, and were structured to explore test content and response process validity.

To inform the focus groups, participants completed the PAQ-A. The PAQ-A is a 7-day recall questionnaire appropriate for adolescents in the school system. It is scored on a 5-point Likert scale and generates a summary score as an indicator of habitual PA levels. The summary score calculation, questionnaire items and format are described in detail elsewhere [31]. Whilst completing the questionnaire, participants were asked (1) to note down any activities that they participated in themselves that were not covered in the questionnaires, (2) to highlight activities they thought were not relevant to them or other people their age, and (3) to highlight any words or activities they did not recognise, understand, or did not think relevant to them. Participants were then able to use these notes or annotations when corresponding questions were posed by the interviewer. Once the PAQ-A was complete, focus groups were conducted by the first author in groups of between three and six participants. The facilitation of the focus group allowed individual representation of views that were derived from previous individual completion of the PAQ-A. The focus groups sought representation of these responses and explored consensus. Focus group questions and terminology were designed in consultation with a Chartered Sport and Exercise Psychologist to address the parameters of test content and response process validity set out by Mâsse and Niet [22]. A flip chart was used to systematically display questions and note responses to allow for children with varying comprehension, competence and attention spans [30, 32]. All focus groups (*n* = 5) took place on school sites, in a suitably quiet area where the activity could be overlooked but not overheard to ensure compliance with safeguarding practice within each school. All focus groups were recorded using a digital audio recorder and ranged from 16–19 min duration (mean 18.5 min).

#### Focus group analysis

Focus groups were transcribed verbatim and transcripts were imported into NVivo 8.0 software and read and re-read to allow familiarisation of the data. The authors followed the protocols associated with that of constructing pen profiles to represent the key and emergent themes from the data (see Mackintosh et al. [32] and Boddy et al. [33]). In summary, the transcripts were analysed using a manual deductive approach followed by an inductive content analysis protocol [32, 33]. Using a combination of the validity criteria proposed by Mâsse and Niet [22] and the focus group guide, themes were categorised through a deductive approach. Emerging themes were inductively created and categorised from data that did not fit the pre-determined categories. Data were then organised schematically to assist with interpretation of the higher and lower order themes. A process of triangular consensus between the authors was employed to inform the credibility and trustworthiness of the results using a reverse tracking process from the pen profiles back to the original transcripts. Finally, an independent researcher, who was not involved in the project, also analysed the data using this technique and provided alternative interpretations, which were then discussed until a consensus had been reached. Through verbatim transcription of data and the triangular consensus procedure described, an agreed acceptable level of credibility and transferability was reached. Due to the breadth of data regarding the understanding of the PAQ-A, a table was deemed more suitable for presenting the data in this case, but it adopted the same processes as used with the pen profiles. Pen profiles can be found in Additional files 1 and 2. Frequency counts refer to the number of focus groups (total focus groups *n* = 5) and example verbatim quotes (with participant number) are included in the pen profiles.

### Phase two

#### Participants

Five secondary schools in North West England were invited to participate. Schools participating in this phase were different to those participating in phase one but were largely representative of schools in the North West in terms of size and academic performance. A total of 177 adolescents aged 11–17 years (44 % female, 94 % White British) provided written informed parental consent and assent.

#### Procedure

Stature and body mass were measured for all participants using standard techniques [34]. Stature was measured using a Leicester Height Measure (Seca Ltd, Birmingham, England) to the nearest 0.1 cm and body mass was measured using calibrated analogue scales (Seca Ltd, Birmingham, England) to the nearest 0.1 kg. Demographic data collected included age, gender and ethnicity. To investigate criterion validity participants wore a uniaxial accelerometer (GT1M model, ActiGraph Ltd, FL, USA) on the right hip for seven consecutive days during waking hours. Accelerometers are reliable and accurate for measuring PA in adolescents [15]. Participants were instructed to complete a log book over the seven days to record the times they wore the accelerometers and describe non-wear time activities. After seven days, the research team collected the accelerometers and participants completed the modified PAQ-A. Participants then completed the modified PAQ-A again approximately 14 days after completing the first questionnaire to assess test-retest reliability [35].

To investigate construct validity the International Fitness Scale (IFIS) [36], measuring perceived fitness levels, and the PA self-efficacy scale (PASES) [37], assessing self-efficacy for engaging in PA, were also administered after seven days alongside the modified PAQ-A. The IFIS comprises five Likert scale questions assessing five key components of fitness (overall fitness, cardiorespiratory fitness, muscular strength, speed and agility, flexibility), which are used to generate a fitness summary score. The PASES is a 17 item questionnaire where respondents are asked whether they agree or disagree with statements relating to three factors: their support-seeking for PA, barriers to PA and positive alternatives to PA. A summary score was calculated by summing the frequency of items that respondents agreed with, then subtracting the frequency of items that were disagreed with. ‘Don’t know’ responses were treated as neutral. All questionnaires were administered through an online platform using school computers during the school day. Trained researchers administered the measurements in schools and supported questionnaire completion.

#### Data management

Accelerometer data were treated in Actilife v.6 (ActiGraph LLC, FL, USA). Time sedentary and in MVPA were determined using age-appropriate accelerometer count cut points [38, 39]. Bouts of 20 min or more of continuous zero counts were excluded from the data and considered as non-wear time [40]. To be included within analyses, participants were required to provide a minimum of 10 h of valid wear time on any three days or more [41]. A daily average was calculated for time spent sedentary, in MVPA and total PA. Modified PAQ-A summary scores were calculated according to published guidelines [31] incorporating the additional questions reported in phase one into the equation. BMI status was determined based on International Obesity Task Force (IOTF) BMI cut points [42].

#### Statistical analysis

Accelerometry variables, IFIS and PASES scores were not normally distributed and were not influenced by transformation methods. These variables are expressed as median and interquartile range. Internal consistency and inter-item reliability were assessed using Cronbach’s coefficient alpha and item-total correlations. Modified PAQ-A test-retest reliability was assessed using intra-class correlation coefficients (ICCs). Spearman rank correlation coefficients between modified PAQ-A scores, MVPA, total PA, IFIS scores, and PASES scores were used to investigate criterion and construct validity. All analyses were conducted using SPSS version 21 (IBM Corp, Armonk, NY) and an alpha level *p* ≤ 0.05 was used to determine significance.

## Results

### Phase one

Participants were 55 % female and 85 % White British, which is largely representative of the North West England (88.4 % White) [43]. Mean age ± standard deviation (SD) of the sample was 14.8 ± 0.7 years. In this sample, the PAQ-A was not adequately representative of all the activities undertaken by participants. For question one, which listed various typical sports and activities, missing (e.g., rounders) and irrelevant activities (e.g., cross-country skiing) were identified in all focus groups (*n* = 5). Irrelevant activities were typically deemed so because of a lack of popularity but also because they were not age-appropriate. As an example:

“Skipping, that’s like for 6 year olds” Girl (G) 3

Missing domains of activity were also identified in focus groups. The before-school period and travel to and from school were highlighted as domains of activity that were not covered in the questionnaire. For example:

“Like there’s no like stuff (questions in the PAQ-A) on before school” G 14

The response process validity evidence revealed common misconceptions, particularly in the respondents’ understanding of what they were being asked to recall. Questions that referred to specific time frames, including questions about after school, evenings, and weekends, were either misinterpreted or there was considerable discrepancy about the time frames in question. These issues were raised in all focus groups. For example:

“It [the questionnaire] says right after school. What does that mean? Right after school?” G 11

Results from phase one indicated that contextual and population or cultural-specific modifications were required before the PAQ-A could be administered in young people of similar socio-cultural background to the present sample. Accordingly, modifications to the questionnaires were made based on the evidence gathered. Potential changes were repeatedly discussed between the authors until consensus had been reached and agreed. Discussions were based on whether the inclusion or exclusion of an item would strengthen the measure of the construct being assessed. Where disagreement occurred, the authors reverted to the literature, exploring this evidence before arriving at consensus whereby the proposed modification was either included or rejected. Specifically, a series of commonly played sports and activities, as reported in the focus groups, were added to question one of the PAQ-A (e.g., tennis, rugby, and cricket). Equally, several irrelevant sports and activities (e.g., cross-country skiing, ringette and street hockey) were removed. A small number of modifications were also made to the terminology used where there were clear cultural differences (e.g., ‘football’ instead of ‘soccer’). All other items in the questionnaire were deemed relevant so no further questions were removed. However, it was suggested that the before-school period and school travel were relevant, but that these domains were not currently represented in the questionnaire. Therefore, questions about the before-school period and active school travel in the morning and afternoon were added (see Additional files 3 and 4). Additional questions were ordered sequentially to aid recall, designed with age appropriate terminology, and were developed using the same format and response options as the original questionnaire. Furthermore, time-specific information was added to questions referring to specific periods of the day. A time frame of “from your last lesson until 6.30 pm” was used to clarify the after-school time period [44]. Time frames were also added to questions on evening and weekend activity. The modified version of the PAQ-A (see Additional file 4) was implemented in phase two.

### Phase two

Demographics, stature and body mass of the sample are shown in Tables 1 and 2 displays the main outcome variables, including self-reported and objectively measured PA. A total of 169 participants (43 % female) completed the modified PAQ-A at the first time point. Those not completing the modified PAQ-A at this point were excluded from all analyses. Participants also completing the IFIS (*n* = 151; 36 % female) and PASES (*n* = 143; 33 % female) were slightly lower than that of the modified PAQ-A owing to technical difficulties accessing the online questionnaires. Those with complete data for these questionnaires were included in the analyses exploring construct validity. Participants who provided valid accelerometry and questionnaire data at the first time point were included in the analysis of criterion validity (*n* = 88). Participants who completed the modified PAQ-A at both time points (*n* = 112) provided evidence for test-retest reliability.

**Table 1:** Descriptive characteristics (mean ± SD)

	Male (*n* = 96)	Female (*n* = 73)
Age (years)	14.2 ± 1.7	14.7 ± 1.6
Ethnicity (% White British)	92.9	97.4
Stature (m)	1.7 ± 0.1	1.6 ± 0.1
Body mass (kg)	55.8 ± 13.0	58.3 ± 11.1
% Healthy weight	80.2	68.5
% Overweight/Obese	19.8	31.5

**Table 2:** Modified PAQ-A scores, objectively measured physical activity, IFIS, and PASES scores

Variable	n	mean ± SD	Minimum	Maximum
Modified PAQ-A				
Full sample	169	2.8 ± 0.6	1.4	4.4
Male	96	2.8 ± 0.6	1.4	4.4
Female	73	2.8 ± 0.6	1.7	4.0
IFIS score^a^	151			
Full sample	151	3.6 [3.2–4.2]	1.0	5.0
Male	96	3.8 [3.2–4.4]	1.0	5.0
Female	55	3.6 [3.0–3.8]	2.4	4.6
PASES score^a^				
Full sample	143	13.0 [9–16]	–17.0	17.0
Male	96	13.0 [8.3–15.8]	–17.0	17.0
Female	47	13.0 [9.0–16.0]	3.0	17.0
Accelerometry				
Total daily PA (mins/d)^a^				
Full sample	88	221.6 [193.1–241.6]	-	-
Male	51	229.8 [199.0–255.5]	-	-
Female	37	198.8 [188.6–231.1]	-	-
Daily MVPA (mins/d)^a^				
Full sample	88	57.1 [42.2–71.0]	-	-
Male	51	58.4 [45.7–73.5]	-	-
Female	37	52.7 [39.8–67.2]	-	-
Daily sedentary time (mins/d)^a^				
Full sample	88	484.5 [458.6–515.7]	-	-
Male	51	487.3 [464.4–522.3]	-	-
Female	37	473.6 [454.5–509.4]	-	-

^a^presented as median and interquartile range

#### Reliability

Test-retest reliability of the modified PAQ-A score was strong (ICC = 0.78, 95 % CI, 0.70, 0.84), as shown in Table 3. ICCs were lower for individual items and ranged between 0.47 and 0.69. Question 9 regarding weekend activity had the lowest test-retest reliability and question 3, which was an added question regarding active travel to school had the highest. Cronbach’s alpha coefficient for the modified PAQ-A score showed acceptable inter-item reliability (α = 0.72). Item-total correlations show how well each item correlates with the composite of the remaining items; correlations ranged from α = 0.24 to 0.54 with all additional and modified questions exceeding α = 0.30.

**Table 3:** Test-retest, internal consistency and inter-item reliability of the modified PAQ-A

	Test-retest reliability	Inter-item reliability	
	ICC (95 % CI)	Cronbach’s alpha coefficient	Corrected item-total correlations
Modified PAQ-A score	0.78 (0.70, 0.84)	0.72	-
Modified question 1. Sport and activity list	0.56 (0.42, 0.67)	-	0.43
Added question 2. Before school activity	0.52 (0.40, 0.64)	-	0.36
Added question 3. To school active travel	0.69 (0.58, 0.77)	-	0.30
Question 4. P.E	0.67 (0.56, 0.76)	-	0.29
Question 5. Lunch	0.64 (0.52, 0.74)	-	0.24
Modified question 6. After school	0.58 (0.44, 0.69)	-	0.56
Added question 7. From school active travel	0.57 (0.44, 0.68)	-	0.33
Modified question 8. Evenings	0.62 (0.50, 0.72)	-	0.39
Modified question 9. Weekend	0.47 (0.32, 0.60)	-	0.39
Question 10. Statement	0.53 (0.39, 0.65)	-	0.54
Question 11. Weekly activity	0.66 (0.55, 0.75)	-	0.53

### Criterion validity

Table 4 presents the associations between the modified PAQ-A scores and the accelerometry variables, namely daily MVPA and total daily PA. The modified PAQ-A scores moderately correlated with MVPA (*r* = 0.39) and total PA (0.42). Question two (an added question) regarding before school activity and question five regarding lunchtime activity were very weakly correlated with accelerometry-derived PA variables. All other questions, including added or modified items, had weak-to-moderate correlations with with daily MVPA, total daily PA or both.

**Table 4:** Spearman correlation coefficients of modified PAQ-A score and PAQ-A questions with objectively measured physical activity and self-reported fitness and PA self-efficacy

	Daily MVPA	Total daily PA	IFIS scores	PASES scores
Modified PAQ-A score	0.39**	0.42**	0.35**	0.32**
Modified question 1. Sport and activity list	0.12	0.21*	0.37**	0.25**
Added question 2. Before school activity	−0.02	0.14	0.20*	0.04
Added question 3. To school active travel	0.32**	0.33**	−0.11	0.02
Question 4. P.E	0.25*	0.12	0.25*	0.10
Question 5. Lunch	−0.04	0.15	0.13	0.05
Modified question 6. After school	0.26*	0.26*	0.18*	0.18*
Added question 7. From school active travel	0.30**	0.22*	−0.07	0.05
Modified question 8. Evenings	0.23*	0.23*	0.39**	0.28**
Modified question 9. Weekend	0.10	0.28**	0.30*	0.29**
Question 10. Statement	0.38**	0.33**	0.40**	0.33**
Question 11. Weekly activity	0.34**	0.29**	0.55**	0.43**

*p < 0.05,**p < 0.01

### Construct validity

The associations between modified PAQ-A scores and self-reported fitness and PA self-efficacy are presented in Table 4. The modified PAQ-A scores were moderately correlated with IFIS (*r* = 0.35) and PASES (*r* = 0.32) scores. Question two was very weakly associated with accelerometry variables but was significantly correlated with IFIS scores (*r* = 0.20). Questions three and seven, both on active travel, were very weakly correlated with IFIS and PASES scores. Question five, which was very weekly correlated with accelerometry variables, was also very weakly associated with IFIS or PASES scores.

## Discussion

The aims of this study were firstly to examine the validity of the original PAQ-A based on test content and response process evidence gathered from focus groups; and secondly to assess the validity and reliability of a modified PAQ-A with English youth. Focus group findings indicated that the content of the original PAQ-A failed to adequately measure the constructs being assessed in this sample, thus contextual amendments were required. Weak to moderate associations were observed between the modified PAQ-A and accelerometry-derived PA, self-reported fitness, and PA self-efficacy, demonstrating acceptable criterion and construct validity. The modified PAQ-A also demonstrated acceptable inter-item and test-retest reliability.

The moderate correlations observed between the modified PAQ-A score and objectively assessed daily MVPA and total daily PA are comparable with findings from the original and subsequent PAQ-A validation studies using accelerometers as the criterion measure [12–14]. Janz and colleagues also modified the PAQ-A but reported correlation coefficients of 0.56 and 0.63 between PAQ scores, total PA and MVPA, which are stronger than those reported in the present study [14]. The modifications made by Janz and colleagues involved rewording the PAQ-A so that it could be applied to out of school term time, which was not a modification that we considered as our data collection occurred in term time. Janz et al. also excluded items not significantly correlated with accelerometry variables, which improved the validity of the PAQ score [14]. Retrospectively removing the morning activity and lunchtime activity questions may have improved the validity of the modified PAQ-A score in the present study; however, the phase one focus group data suggested these were relevant domains and therefore we refrained from this approach in this study.

Fitness is a strong indicator of habitual PA in young people [45] and therefore the moderate correlation with self-reported fitness provides encouraging construct validity evidence for the modified PAQ-A. This is consistent with previous research also reporting moderate correlations between the PAQ-C and objectively measured fitness [16]. Construct validity is further supported by the observed associations with PA self-efficacy. PA self-efficacy is an established correlate of youth PA [46–48]. Original data presented moderate correlations with perceived athletic competence [16], which has similar characteristics to PA self-efficacy in relation to young people’s beliefs that they feel enabled to engage in PA [49].

We observed that the modified PAQ-A score was stable over time (ICC = 0.78), which is comparable with original research [17] and subsequent reliability studies [13, 14]. Cronbach’s alpha coefficients showed acceptable internal consistency, similar to the original and other modified versions of the PAQ-A [13, 14, 17]. Item-total correlations were high for added and modified questions, but correlations for original questions on lunchtime and physical education were low, which is consistent with previous findings [10, 13, 14]. Questions that asked respondents to report overall activity levels (questions 10 and 11) as opposed to specific times showed higher internal consistency compared to questions asking respondents to recall specific time frames. A plausible explanation for this finding may be that activity during specific time frames varies day by day. These data confirm the validity and reliability of the modified PAQ-A.

The major strength of this study is the mixed methodological approach, adopting a formative aspect followed by a quantitative component. The methodological rigour employed throughout ensured credibility and transferability of the findings. Focus groups or interviews are deemed appropriate for obtaining content validity evidence [22, 29], but quantitative approaches are commonly used [10, 50]. For example, Bervoets and colleagues employed an expert panel to review questionnaire items and rate the relevance of each item to the underlying construct, from which numerical scores were generated [10]. Focus groups, on the other hand, have the additional benefit of being able to target the intended respondents themselves, providing evidence that may have been overlooked with alternative approaches. The facilitation of focus groups allowed individual representation of views whilst simultaneously seeking consensus. Together with the second phase, this study provides a robust process for examining the validity and reliability of the PAQ-A.

A potential limitation of this study is the use of self-reported fitness, which may have introduced response bias. Factors such as social desirability may have led to an overestimation of fitness levels. Furthermore, construct validity cannot truly be established without evidence of divergent validity, which has not been determined in this study. Further studies to strengthen the validity evidence for the modified PAQ-A may include a measure to examine divergent validity alongside an alternative measure of construct validity, such as objectively measured fitness. While we acknowledge this as a limitation, the IFIS has shown acceptable validity in youth populations [36]. In addition, the reliability of the modified PAQ-A may have been underestimated due to natural variation in physical activity levels from the first test to retest.

Prior testing of the modified PAQ-A in a small sample of adolescents before phase two may have provided new information to further refine the questionnaire. However, phase two in itself has served this purpose. Subsequent studies may indeed adapt the modified version of the PAQ-A further. For example, removal of the morning and/or lunchtime activity would have improved criterion validity and therefore could be omitted in future use in this population.

## Conclusion

The original PAQ-A required contextual and population/cultural-specific modifications to inform a representative version for adolescents from North West England. Although further testing with more diverse English samples is required, the modified PAQ-A appears to demonstrate acceptable validity and reliability. Similar formative approaches examining the suitability of questionnaires should be applied in future research.

## Additional files

Additional file 1:
**Test content validity evidence for the PAQ-A.** (DOCX 46 kb) Additional file 2:
**Response processes validity evidence for the PAQ-A.** (DOCX 19 kb) Additional file 3:
**Questionnaire modifications and rationale.** (DOC 33 kb) Additional file 4:
**Physical activity questionnaire.** (PDF 183 kb)

## References

[1] Strong WB, Malina RM, Blimkie CJ, Daniels SR, Dishman RK, Gutin B (2005). Evidence based physical activity for school-age youth. J Pediatr.

[2] Scholes S, Mindell J. Physical activity in children (Ch 3). Health Survey for England 2012 annual report. 2013. http://www.hscic.gov.uk/catalogue/PUB13218.

[3] Voss C, Ogunleye AA, Sandercock GR (2013). Physical Activity Questionnaire for children and adolescents: English norms and cut-off points. Pediatr Int.

[4] Sallis JF, Saelens BE (2000). Assessment of physical activity by self-report: status, limitations, and future directions. Res Q Exercise Sport.

[5] Chinapaw MJM, Mokkink LB, van Poppel MNM, van Mechelen W, Terwee CB (2010). Physical Activity Questionnaires for Youth A Systematic Review of Measurement Properties. Sports Med.

[6] Kowalski KC, Crocker PRE, Donen RM (2004). The Physical Activity Questionnaire for Older Children (PAQ-C) and Adolescents (PAQ-A) Manual.

[7] Biddle SJ, Gorely T, Pearson N, Bull FC (2011). An assessment of self-reported physical activity instruments in young people for population surveillance: Project ALPHA. Int J Behav Nutr Phys Act.

[8] Adeniyi AF, Okafor NC, Adeniyi CY (2011). Depression and physical activity in a sample of nigerian adolescents: levels, relationships and predictors. J. Child Adolesc. Psychiatr. Ment. Health.

[9] Asare M, Danquah SA (2015). The relationship between physical activity, sedentary behaviour and mental health in Ghanaian adolescents. J. Child Adolesc. Psychiatr. Ment. Health.

[10] Bervoets L, Van Noten C, Van Roosbroeck S, Hansen D, Van Hoorenbeeck K, Verheyen E (2014). Reliability and Validity of the Dutch Physical Activity Questionnaires for Children (PAQ-C) and Adolescents (PAQ-A). Arch Public Health.

[11] Maher CA, Williams MT, Olds T, Lane AE (2007). Physical and sedentary activity in adolescents with cerebral palsy. Dev Med Child Neurol.

[12] Kowalski KC, Crocker PRE, Kowalski NP (1997). Convergent validity of the physical activity questionnaire for adolescents. Pediatr Exerc Sci.

[13] Martinez-Gomez D, Martinez-de-Haro V, Pozo T, Welk GJ, Villagra A, Calle ME (2009). Reliability and validity of the PAQ-A questionnaire to assess physical activity in Spanish adolescents. Rev Esp Salud Publica.

[14] Janz KF, Lutuchy EM, Wenthe P, Levy SM (2008). Measuring activity in children and adolescents using self-report: PAQ-C and PAQ-A. Med Sci Sports Exerc.

[15] Trost SG (2007). State of the Art Reviews: Measurement of Physical Activity in Children and Adolescents. Am J Lifestyle Med.

[16] Kowalski KC, Crocker PRE, Faulkner RA (1997). Validation of the physical activity questionnaire for older children. Pediatr Exerc Sci.

[17] Crocker PR, Bailey DA, Faulkner RA, Kowalski KC, McGrath R (1997). Measuring general levels of physical activity: preliminary evidence for the Physical Activity Questionnaire for Older Children. Med Sci Sports Exerc.

[18] Mundt CA, Baxter-Jones AD, Whiting SJ, Bailey DA, Faulkner RA, Mirwald RL (2006). Relationships of activity and sugar drink intake on fat mass development in youths. Med Sci Sports Exerc.

[19] Baxter-Jones AD, Kontulainen SA, Faulkner RA, Bailey DA (2008). A longitudinal study of the relationship of physical activity to bone mineral accrual from adolescence to young adulthood. Bone.

[20] Aggio D, Ogunleye AA, Voss C, Sandercock GR (2012). Temporal relationships between screen-time and physical activity with cardiorespiratory fitness in English schoolchildren: a 2-year longitudinal study. Prev Med.

[21] Biddle SJ, Gorely T, Pearson N, Bull FC (2011). An assessment of self-reported physical activity instruments in young people for population surveillance: Project ALPHA. Int J Behav Nutr Phys Act.

[22] Masse LC, de Niet JE (2012). Sources of Validity Evidence Needed With Self-Report Measures of Physical Activity. J Phys Act Health.

[23] Stamatakis E, Chaudhury M (2008). Temporal trends in adults’ sports participation patterns in England between 1997 and 2006: the Health Survey for England. Br J Sports Med.

[24] Terwee CB, Mokkink LB, van Poppel MN, Chinapaw MJ, van Mechelen W, de Vet HC (2010). Qualitative attributes and measurement properties of physical activity questionnaires: a checklist. Sports Med.

[25] Voss C, Sandercock G, Higgins JW, Macdonald H, Nettlefold L, Naylor PJ (2014). A cross-cultural comparison of body composition, physical fitness and physical activity between regional samples of Canadian and English children and adolescents. Can J Public Health.

[26] Bervoets L, Van Noten C, Van Roosbroeck S, Hansen D, Van Hoorenbeeck K, Verheyen E (2014). Reliability and Validity of the Dutch Physical Activity Questionnaires for Children (PAQ-C) and Adolescents (PAQ-A). Archives Public Health.

[27] Moore JB, Hanes JC, Barbeau P, Gutin B, Trevino RP, Yin ZN (2007). Validation of the Physical Activity Questionnaire for Older Children in children of different races. Pediatr Exerc Sci.

[28] Dopp RR, Mooney AJ, Armitage R, King C (2012). Exercise for adolescents with depressive disorders: a feasibility study. Depress Res Treat.

[29] Goodwin LD, Leech NL (2003). The meaning of validity in the new Standards For Educational and Psychological Testing: Implications for measurement courses. Meas Eval Couns Dev.

[30] Porcellato L, Dughill L, Springett J (2002). Using focus groups to explore children’s perceptions of smoking: reflections on practice. Health Educ.

[31] Kowalski K, Crocker R, Donen R (2004). The Physical Activity Questionnaire for Older Children (PAQ-C) and Adolescents (PAQ-A) Manual.

[32] Mackintosh KA, Knowles ZR, Ridgers ND, Fairclough SJ. Using formative research to develop CHANGE!: a curriculum-based physical activity promoting intervention. BMC Public Health. 2011;11. 10.1186/1471-2458-11-831 PMC3214189 22032540

[33] Boddy LM, Knowles ZR, Davies IG, Warburton GL, Mackintosh KA, Houghton L et al. Using formative research to develop the healthy eating component of the CHANGE! school-based curriculum intervention. BMC Public Health. 2012;12. 10.1186/1471-2458-12-710 PMC3548762 22931457

[34] Lohman TG, Roche AF, Martorell R (1988). Anthropometric standardization reference manual.

[35] Marx RG, Menezes A, Horovitz L, Jones EC, Warren RE (2003). A comparison of two time intervals for test-retest reliability of health status instruments. J Clin Epidemiol.

[36] Ortega FB, Ruiz JR, Espana-Romero V, Vicente-Rodriguez G, Martinez-Gomez D, Manios Y (2011). The International Fitness Scale (IFIS): usefulness of self-reported fitness in youth. Int J Epidemiol.

[37] Saunders RP, Pate RR, Felton G, Dowda M, Weinrich MC, Ward DS (1997). Development of questionnaires to measure psychosocial influences on children’s physical activity. Prev Med.

[38] Evenson KR, Catellier DJ, Gill K, Ondrak KS, McMurray RG (2008). Calibration of two objective measures of physical activity for children. J Sports Sci.

[39] Trost SG, Loprinzi PD, Moore R, Pfeiffer KA (2011). Comparison of accelerometer cut points for predicting activity intensity in youth. Med Sci Sports Exerc.

[40] Masse LC, Fuemmeler BF, Anderson CB, Matthews CE, Trost SG, Catellier DJ (2005). Accelerometer data reduction: a comparison of four reduction algorithms on select outcome variables. Med Sci Sports Exerc.

[41] Rich C, Geraci M, Griffiths L, Sera F, Dezateux C, Cortina-Borja M (2013). Quality Control Methods in Accelerometer Data Processing: Defining Minimum Wear Time. PLoS One.

[42] Cole TJ, Bellizzi MC, Flegal KM, Dietz WH (2000). Establishing a standard definition for child overweight and obesity worldwide: international survey. Brit Med J.

[43] Office For National Statistics (2011). Population Estimates by Ethnic Group 2002 – 2009. http://www.ons.gov.uk/ons/taxonomy/index.html?nscl=Population+Estimates+by+Ethnic+Group. Accessed 30/06/2015.

[44] Atkin AJ, Gorely T, Biddle SJH, Marshall SJ, Cameron N (2008). Critical Hours: Physical Activity and Sedentary Behavior of Adolescents After School. Pediatr Exerc Sci.

[45] Aires L, Andersen LB, Mendonca D, Martins C, Silva G, Mota J (2010). A 3-year longitudinal analysis of changes in fitness, physical activity, fatness and screen time. Acta Paediatr.

[46] Van Der Horst K, Paw MJ, Twisk JW, Van Mechelen W (2007). A brief review on correlates of physical activity and sedentariness in youth. Med Sci Sports Exerc.

[47] Hearst MO, Patnode CD, Sirard JR, Farbakhsh K, Lytle LA (2012). Multilevel predictors of adolescent physical activity: a longitudinal analysis. Int J Behav Nutr Phys Act.

[48] Craggs C, Corder K, van Sluijs EM, Griffin SJ (2011). Determinants of change in physical activity in children and adolescents: a systematic review. Am J Prev Med.

[49] Welk GJ (1999). The Youth Physical Activity Promotion Model: A conceptual bridge between theory and practice. Quest.

[50] Dunn JGH, Bouffard M, Rogers WT (1999). Assessing Item Content-Relevance in Sport Psychology Scale-Construction Research: Issues and Recommendations. Meas Phys Educ Exerc Sci.

