# Supplementary material for: Validity and reliability of a modified english version of the physical activity questionnaire for adolescents
Source: Arch Public Health. 2016 Jan 22;74:3. doi: 10.1186/s13690-016-0115-2 (PMC4724149; doi:10.1186/s13690-016-0115-2)
Supplement: Additional file 1: — Test content validity evidence for the PAQ-A. (DOCX 46 kb) [file 13690_2016_115_MOESM1_ESM.docx]

**Test content validity evidence for the PAQ-A.**

B = Adolescent Boy. G = Adolescent Girl.

**Relevant activities** (n=2)

*“Not everyone would be able to do everything but mostly it was like, well, suited for most people.”* B1

**Irrelevant activities** (n=5)*

*“Skipping, that's like for six year olds”* G3

**Misunderstood** (n=1)

Understanding of the term ‘very active’: *“Like if you just like were you concentrating on what you’re just doing.”* G3

**Understood** (n=5)

Understanding of the term ‘very active’: *“Where you actually get out of breath and stuff or actually feel a bit tired”* B5

**Missing activities** (n=5)*

*“Rounder’s, because we do it in school.”* G11

**Relevant domains** (n=4)

*“It covers all the aspects of physical”* B6

**Missing domains** (n=4)*

*“Like I say before school if you’re doing anything”* G4

**Understanding of terminology**

**PAQ -- Test Content Validity**

**Relevance of activities**

**Relevance of domains of activity**
