# Supplementary material for: Validity and reliability of a modified english version of the physical activity questionnaire for adolescents
Source: Arch Public Health. 2016 Jan 22;74:3. doi: 10.1186/s13690-016-0115-2 (PMC4724149; doi:10.1186/s13690-016-0115-2)
Supplement: Additional file 2: — Response processes validity evidence for the PAQ-A. (DOCX 19 kb) [file 13690_2016_115_MOESM2_ESM.docx]

| **RESPONSE PROCESSES (PAQ-A)** | | | |
| --- | --- | --- | --- |
| **Able to recall information**  **Response processes validity evidence for the PAQ-A.** | Accurate recall (n=5):  *“It’s fairly easy to remember that really.”* B2 | Recall issues (n=4):  *“I think the whole week period that the questions are based on seems a bit long.”* B3 |  |
| **Format** | Positive (n=4):  *“Quite well set out, easy to understand really.”* B2 | | |
| **Able to answer truthfully** | Yes (n=5):  *“Quite easy because you’re writing it down not like talking to someone”* B4 |  |  |
| **Understanding of questions** | | | |
| *Physical activity in your spare time: Have you done any of the following activities in the past 7 days (last week)? If yes, how many times?* | Understood (n=5):  *“How much exercise have you done in the last seven days”* G4 | Misunderstood (n=1):  *“I’m not too sure really.”* B4 |  |
| *In the last 7 days, during your physical education (PE) classes, how often were you very active (playing hard, running, jumping, throwing)?* | Understood (n=5):  *“During PE lessons, how hard we actually try”* B6 |  |  |
| *In the last 7 days, what did you normally do at lunch (besides eating lunch)?* | Understood (n=5):  *“What you do during lunch if you’re like not eating”* G4 |  |  |
| *In the last 7 days, on how many days right after school, did you do sports, dance, or play games in which you were very active?* | Understood (n=5): *“Like you do a couple of things at school straight after.”* G5 | Misunderstood (n=3):  *“It says right after school. What does that mean? Right after school?”* G11 | Time discrepancies (n=3):  *“Well if you like doing something at 7 o’clock which isn’t right after school*  *is that after school”* G11 |
| *In the last 7 days, on how many evenings did you do sports, dance, or play games in which you were very active?* | Understood (n=4): *“See if you do sports in the evening”* B6 |  | Time discrepancies (n=2): *“5:00. I guess 5:00 or 6:00.”* G9 |
| *On the last weekend, how many times did you do sports, dance, or play games in which you were very active?* | Understood (n=4):  In response to what they included in the weekend:  *“Saturday and Sunday.”* B7 |  | Time discrepancies (n=2):  In response to what they included in the weekend:  *“I’d say Friday night to Sunday morning.”* B4 |
| *Which one of the following describes you best for the last 7 days? Read all five statements before deciding on the one answer that describes you.* | Appropriate option (n=5):  In response to whether the statements were appropriate for them:  *“I think they’re alright”* B5 |  |  |
| *Mark how often you did physical activity (like playing sports, games, doing dance, or any other physical activity) for each day last week.* | Understood (n=5): *“Like how much we can take part in everyday”* G11 | Misunderstood (n=1):  *“What does it mean? Does it mean counting in your PE lessons or is it just in your free time?”* G1 |  |

B = Adolescent Boy. G = Adolescent Girl.
