# Supplementary material for: Validity and reliability of a modified english version of the physical activity questionnaire for adolescents
Source: Arch Public Health. 2016 Jan 22;74:3. doi: 10.1186/s13690-016-0115-2 (PMC4724149; doi:10.1186/s13690-016-0115-2)
Supplement: Additional file 3: — Questionnaire modifications and rationale. (DOC 33 kb) [file 13690_2016_115_MOESM3_ESM.doc]

| **Original version**  **Questionnaire modifications and rationale** | **Amendment made** | **Modified version** | **Rationale** |
| --- | --- | --- | --- |
| **PAQ-A** | | | |
| Question 1 list of activities (see original) | Removal and addition of listed activities | Specific activities were removed and some added to those listed. See modified questionnaire in supplementary files. | All focus groups highlighted missing activities and activities deemed irrelevant compared to what they normally do or expect people their age participate in. |
| Question: In the last 7 days, on how many days *right after school*, did you do sports, dance, or play games in which you were very active? (Check one only.) | Addition of contextual information | In the last 7 days, on how many days right after school **(from your last lesson until**  **6.30pm),** did you do sports, dance, or play games in which you were very active? (Tick one only.) | Three focus groups demonstrated an overall lack of understanding with this question and there were varied interpretations of the time period in question. Contextual information added to clarify. |
| Question: In the last 7 days, on how many *evenings* did you do sports, dance, or play games in which you were very active? (Check one only.) | Addition of contextual information | In the last 7 days, on how many evenings **(from 6.30pm until you go to bed)** did you do sports, dance, or play games in which you were very active? (Tick one only.) | Three focus groups reported various interpretations of the time period in question. Contextual information added to clarify the time in question. |
| Question: *On the last weekend*, how many times did you do sports, dance, or play games in which you were very active? (Check one only.) | Addition of contextual information | On the last weekend **(Saturday and Sunday)**, how many times did you do sports,  dance, or play games in which you were very active? (Tick one only.) | Two focus groups demonstrated various interpretations of the time period in question. Contextual information added to clarify the time in question. |
| N/A | Added question | In the last 7 days, on how many days before school, did you do sports, dance, or  play games in which you were very active? (Tick one only.) | Two focus groups highlighted the before school period as a period of time in which they are active, but this is not represented in the questionnaire. Additional question added for this time period. |
| N/A | Added question | In the last 7 days, on how many mornings did you actively travel (for example,  walking, cycling, scooting and skateboarding) to school? (Tick one only.) | One focus group highlighted the journey to and from school as a period of time in which they are active but that was not represented in the questionnaire. |
| N/A | Added question | In the last 7 days, on how many afternoons did you actively travel (for example,  walking, cycling, scooting and skateboarding) from school? (Tick one only.) | As above. |
