# Supplementary material for: Validity and reliability of a modified english version of the physical activity questionnaire for adolescents
Source: Arch Public Health. 2016 Jan 22;74:3. doi: 10.1186/s13690-016-0115-2 (PMC4724149; doi:10.1186/s13690-016-0115-2)
Supplement: Additional file 4: — Physical activity questionnaire. (PDF 183 kb) [file 13690_2016_115_MOESM4_ESM.pdf]

PHYSICAL ACTIVITY QUESTIONNAIRE

On this page, we are trying to find out about your level of physical activity from the last 7 days (in the last week). This includes sports or dance that make you sweat or make your legs feel tired, or games that make you breathe hard, like tag, skipping, running, climbing, and others.

Remember:
1. There are no right and wrong answers — this is not a test.
2. Please answer all the questions as honestly and accurately as you can — this is very important.

*1. Physical activity in your spare time: Have you done any of the following activities in the past 7 days (last week)? If yes, how many times? (Tick only one circle per row.)

	No	1-2	3-4	5-6	7+
Skipping	○	○	○	○	○
Rowing/canoeing	○	○	○	○	○
Walking for exercise	○	○	○	○	○
Cycling	○	○	○	○	○
Jogging or running	○	○	○	○	○
Aerobics	○	○	○	○	○
Swimming	○	○	○	○	○
Football	○	○	○	○	○
Baseball, softball	○	○	○	○	○
Dance	○	○	○	○	○
Badminton	○	○	○	○	○
Skateboarding	○	○	○	○	○
Volleyball	○	○	○	○	○
Floor hockey	○	○	○	○	○
Basketball	○	○	○	○	○
Ice skating	○	○	○	○	○
Ice hockey	○	○	○	○	○
Trampolining	○	○	○	○	○
Boxing	○	○	○	○	○
Gym activities	○	○	○	○	○
Gymnastics	○	○	○	○	○
Netball	○	○	○	○	○
Rounders	○	○	○	○	○
Rugby	○	○	○	○	○
Tennis	○	○	○	○	○
Cricket	○	○	○	○	○
Other (1)	○	○	○	○	○
Other (2)	○	○	○	○	○

Please list your 'other' activities (for example, other 1 = American football and other 2 = bowls)

***2. In the last 7 days, on how many days before school, did you do sports, dance, or play games in which you were very active? (Tick one only.)**

- ◯ None
- ◯ 1 time last week
- ◯ 2 or 3 times last week
- ◯ 4 times last week
- ◯ 5 times last week

***3. In the last 7 days, on how many mornings did you actively travel (for example, walking, cycling, scootering and skateboarding) to school? (Tick one only.)**

- ◯ None
- ◯ 1 time last week
- ◯ 2 or 3 times last week
- ◯ 4 times last week
- ◯ 5 times last week

***4. In the last 7 days, during your physical education (PE) classes, how often were you very active (playing hard, running, jumping, throwing)? (Tick one only.)**

- ◯ I don’t do PE
- ◯ Hardly ever
- ◯ Sometimes
- ◯ Quite often
- ◯ Always

***5. In the last 7 days, what did you normally do at lunch (besides eating lunch)? (Tick one only.)**

- ◯ Sat down (talking, reading, doing schoolwork)
- ◯ Stood around or walked around
- ◯ Ran or played a little bit
- ◯ Ran around and played quite a bit
- ◯ Ran and played hard most of the time

***6. In the last 7 days, on how many days right after school (from your last lesson until 6.30pm), did you do sports, dance, or play games in which you were very active? (Tick one only.)**

- ◯ None
- ◯ 1 time last week
- ◯ 2 or 3 times last week
- ◯ 4 times last week
- ◯ 5 times last week

***7. In the last 7 days, on how many afternoons did you actively travel (for example, walking, cycling, scootering and skateboarding) from school? (Tick one only.)**

- ◯ None
- ◯ 1 time last week
- ◯ 2 or 3 times last week
- ◯ 4 times last week
- ◯ 5 times last week

***8. In the last 7 days, on how many evenings (from 6.30pm until you go to bed) did you do sports, dance, or play games in which you were very active? (Tick one only.)**

- ◯ None
- ◯ 1 time last week
- ◯ 2 or 3 times last week
- ◯ 4 or 5 last week
- ◯ 6 or 7 times last week

***9. On the last weekend (Saturday and Sunday), how many times did you do sports, dance, or play games in which you were very active? (Tick one only.)**

- ◯ None
- ◯ 1 time
- ◯ 2 — 3 times
- ◯ 4 — 5 times
- ◯ 6 or more times

***10. Which one of the following describes you best for the last 7 days? Read all five statements before deciding on the one answer that describes you.**

- ◯ All or most of my free time was spent doing things that involve little physical effort
- ◯ I sometimes (1 — 2 times last week) did physical things in my free time (e.g. played sports, went running, swimming, bike riding, did aerobics)
- ◯ I often (3 — 4 times last week) did physical things in my free time
- ◯ I quite often (5 — 6 times last week) did physical things in my free time
- ◯ I very often (7 or more times last week) did physical things in my free time

***11. Mark how often you did physical activity (like playing sports, games, doing dance, or any other physical activity) for each day last week.**

	None	Little bit	Medium	Often	Very often
Monday	◯	◯	◯	◯	◯
Tuesday	◯	◯	◯	◯	◯
Wednesday	◯	◯	◯	◯	◯
Thursday	◯	◯	◯	◯	◯
Friday	◯	◯	◯	◯	◯
Saturday	◯	◯	◯	◯	◯
Sunday	◯	◯	◯	◯	◯

***12. Were you ill/injured last week, or did anything prevent you from doing your normal physical activities? (Tick one.)**

- ◯ Yes
- ◯ No

13. If Yes, what prevented you?
